# Integrated Multivariate Analysis and Desirability-Based Optimization of Milk–Whey Mixtures: Effects on Physicochemical Properties, Amino Acid Profile, and Nutritional Quality

**DOI:** 10.3390/foods15101759

**Published:** 2026-05-15

**Authors:** Albina Kaumenova, Dina Dautkanova, Zhanna Dossimova, Zhannur Niyazbekova, Botakoz Seisenbikyzy, Zhulduz Suleimenova, Nurgul Myrzabayeva, Ayazhan Zagypan, Maksat Serikov, Gulmira Kenenbay, Zoltan Kovacs, Flora Vitalis, Assiya Serikbayeva, Maxat Toishimanov

**Affiliations:** 1 Scientific and Educational Innovation Center for Technologies and Quality of Food Products, Kazakh National Agrarian Research University, Almaty 050010, Kazakhstan; 2 Kazakh Scientific Research Institute of Processing and Food Industry, Almaty 050000, Kazakhstangkenenbay@mail.ru (G.K.); 3 Reference Center for Safety and Quality of Agricultural Products, Kazakh National Agrarian Research University, Almaty 050010, Kazakhstan; zhanna.dossimova@kaznaru.edu.kz (Z.D.);; 4 Technology and Food Safety Department, Kazakh National Agrarian Research University, Almaty 050010, Kazakhstan; 5 Food and Environment Safety Laboratory, Kazakh National Agrarian Research University, Almaty 050010, Kazakhstanazaypan@mail.ru (A.Z.); 6 Kazakh Scientific Research Veterinary Institute LLP, Almaty 050016, Kazakhstan; 7 Department of Food Measurements and Process Control, Institute of Food Science and Technology, Hungarian University of Agriculture and Life Sciences, 1118 Budapest, Hungary; 8 Department of Zooengineering and Biotechnology, Kazakh National Agrarian Research University, Almaty 050010, Kazakhstan

**Keywords:** whey valorization, whey–milk mixtures, amino acid composition, physicochemical properties, multivariate analysis, desirability function

## Abstract

The valorization of dairy by-products, particularly whey, represents a key challenge and opportunity in sustainable food systems. This study aimed to evaluate the physicochemical and amino acid composition of milk and whey-derived products and to identify optimal whey–milk mixtures using integrated multivariate and desirability-based approaches. Ten model systems (M1–M10) were prepared with increasing whey content (7.5–75%), and their composition was analyzed using infrared spectroscopy and high-performance liquid chromatography. Multivariate analysis, including PCA and correlation heatmaps, revealed that protein, casein, TS, SNF, and amino acid fractions (ΣEAA and ΣBCAA) were the primary drivers of compositional variability, whereas lactose and acidity-related parameters contributed to secondary differentiation. Desirability function analysis was applied by integrating nutritional quality, functional balance, and sustainability score into a composite index. The results demonstrated that intermediate formulations achieved a more balanced profile compared with extreme compositions. Among all mixtures, the formulation containing 30% whey (M5) showed the highest overall desirability within the evaluated parameters, reflecting a favorable balance between compositional quality and whey utilization. These findings highlight the potential of integrated analytical approaches for the development of nutritionally optimized and resource-efficient dairy systems.

## 1. Introduction

The global dairy industry generates enormous volumes of by-products during the processing of cheese, cottage cheese, and fermented products, with whey representing the most voluminous and environmentally significant fraction. Approximately 9 kg of liquid whey is generated as a by-product for every kilogram of cheese produced, leading to global production volumes exceeding 115 million tonnes annually. Despite its considerable nutritional value—comprising lactose, soluble proteins, lipids, minerals, and bioactive compounds—approximately 47% of cheese whey continues to be discharged directly into water bodies or drainage systems, causing severe ecological consequences including eutrophication, an elevated biochemical oxygen demand of 40–60 g/L, and a chemical oxygen demand of 50–80 g/L. These environmental pressures, combined with increasingly stringent regulatory frameworks in the European Union and other jurisdictions, have intensified scientific and industrial interest in the valorization of dairy by-products within circular bioeconomy paradigms [1,2,3,4,5].

Whey is a nutritionally heterogeneous matrix whose composition depends substantially on the type of primary dairy product from which it derives [6]. Sweet whey, obtained from rennet-coagulated cheese, is compositionally distinct from acid whey recovered during cottage cheese or yogurt production, differing primarily in pH, acidity, lactic acid content, and the degree of casein hydrolysis [7]. On a dry matter basis, lactose constitutes approximately 70% of whey solids, followed by proteins (~14%), minerals (~7–8%), and lipids (~5–6%). Whey proteins, including β-lactoglobulin, α-lactalbumin, serum albumin, and immunoglobulins, represent a distinct functional fraction absent in the residual whey obtained after casein micelle coagulation [8]. The ratio of whey protein to casein in unprocessed bovine milk is approximately 20:80, and its modification—through dilution with whey or selective fractionation—has been demonstrated to significantly alter heat stability, viscosity, gel-forming capacity, and the overall physicochemical performance of the resulting dairy matrix [9,10,11].

The nutritional importance of whey proteins in human health is widely recognized. Whey provides a complete protein source, supplying all nine essential amino acids in physiologically relevant proportions, with particularly high levels of branched-chain amino acids (BCAAs; leucine, isoleucine, and valine). These amino acids play a key role in activating the mechanistic target of rapamycin (mTOR) signaling pathway, thereby promoting muscle protein synthesis, regulating metabolism, and supporting energy balance [12,13]. In particular, leucine functions as the primary anabolic trigger for muscle protein synthesis, while the full complement of EAA is required to sustain the complete synthetic response. The nutritional profile of whey-derived fractions is, however, substantially altered by upstream dairy processing, including fermentation, acidification, and curd formation, processes that modify protein structure, induce proteolysis, and redistribute amino acid fractions between the curd and the serum phase [14]. Understanding these compositional transformations across different whey types is therefore critical for designing evidence-based formulations with optimal nutritional functionality.

From a food technology perspective, the incorporation of whey into milk-based systems represents a promising strategy for simultaneously achieving resource valorization and nutritional optimization. Partial replacement of milk with whey has been investigated in the context of yogurt, dahi, beverage, and functional drink formulations, with findings demonstrating that controlled incorporation of whey can enhance total protein content, increase mineral density, and reduce lactose concentration relative to pure whey, while maintaining acceptable sensory and physicochemical properties [15]. The technological performance of whey–milk blends depends critically on the proportional balance between casein-rich and whey protein-rich fractions, which governs colloidal stability, heat-induced aggregation behavior, and gel network formation. Despite increasing evidence supporting the functional potential of whey–milk mixtures, a comprehensive, multi-parameter evaluation of these blends—covering physicochemical properties, as well as amino acid and fatty acid profiles across a continuous compositional gradient—remains insufficiently explored [16].

The physicochemical characterization of dairy matrices has been increasingly advanced by the application of high-throughput analytical techniques, including Fourier-transform infrared (FTIR) spectroscopy and high-performance liquid chromatography (HPLC) [17]. Infrared spectroscopy, in particular, enables the simultaneous quantification of fat, protein, casein, lactose, total solids, and acidity-related parameters through a single rapid measurement, offering substantial advantages over conventional wet chemistry methods in terms of throughput, reproducibility, and resource efficiency [18,19]. HPLC following phenylisothiocyanate (PITC) derivatization remains the reference method for quantitative amino acid profiling in dairy matrices, enabling the resolution of individual free and total amino acids with high sensitivity. The integrated application of these complementary analytical platforms enables a multi-dimensional compositional characterization that is prerequisite for rigorous dairy matrix differentiation and formulation optimization.

Multivariate statistical methods, such as principal component analysis (PCA) and hierarchical cluster analysis (HCA), are widely used in food science to reduce the dimensionality of complex datasets, identify key sources of variation, and reveal clustering patterns that are not evident from univariate approaches. PCA-based approaches have demonstrated particular utility in milk and dairy research for differentiating product categories, monitoring process-induced changes, and identifying compositional markers of quality and authenticity. Correlation heatmaps complement PCA by revealing the structure of pairwise relationships among compositional variables, thereby facilitating mechanistic interpretation of the observed multivariate patterns. However, the translation of multivariate compositional information into actionable formulation decisions requires additional optimization frameworks capable of integrating multiple simultaneously optimized response variables into a single composite index [20,21].

The desirability function approach, originally proposed by Derringer and Suich, is a well-established multi-response optimization methodology widely applied in food product development and process engineering. In this framework, each individual response is transformed into a dimensionless desirability score ranging from 0 (completely unacceptable) to 1 (fully desirable), and the individual scores are aggregated—typically as a weighted geometric mean—into an overall composite desirability index. The method allows simultaneous optimization of competing or correlated response variables, enabling the identification of formulations that achieve the best global compromise across nutritional, technological, and sustainability-related criteria. The integration of PCA with desirability function optimization represents a methodological advancement over conventional single-variable approaches, as it accounts for both the primary axes of compositional variability and the multi-criteria nature of functional food design [22,23].

Despite extensive research on whey valorization and its incorporation into dairy beverages, most previous studies have focused on specific product types, fermentation processes, or single-response optimization strategies. In particular, whey has been widely investigated as a functional ingredient in beverages and bio-based products due to its nutritional value and sustainability potential. However, a systematic evaluation of milk–whey mixtures across a broad compositional gradient, integrating physicochemical characteristics, amino acid profiles, and multivariate statistical analysis, remains limited [24].

Furthermore, previous approaches to formulation optimization have typically relied on individual parameters or simplified criteria, without incorporating multi-response optimization frameworks that simultaneously account for nutritional quality, functional balance, and sustainability. Given the increasing importance of circular bioeconomy strategies in dairy processing, there is a need for integrated methodologies capable of translating complex compositional data into practical formulation decisions [25].

Therefore, the novelty of the present study lies in the combined application of physicochemical analysis, amino acid profiling, multivariate statistical tools (PCA, correlation analysis), and desirability-based optimization to evaluate and identify optimal milk–whey formulations (cottage cheese whey, cheese whey, and yogurt whey), followed by the systematic evaluation of ten whey–milk mixture formulations prepared across a compositional gradient of 7.5–75% whey content. The primary objectives were: (i) to characterize and differentiate the physicochemical and amino acid composition of milk and whey products using FTIR spectroscopy and HPLC; (ii) to apply multivariate statistical analysis (PCA, HCA, Pearson correlation) to elucidate the principal drivers of compositional variability among dairy matrices; and (iii) to identify the optimal whey–milk mixture using an integrated desirability function approach incorporating nutritional quality, physicochemical balance, and sustainability criteria. The results of this study provide a scientific basis for the evidence-based formulation of sustainable and nutritionally optimized dairy products through the valorization of whey by-products.

## 2. Materials and Methods

### 2.1. Raw Materials

Raw cow’s milk was obtained from a commercial dairy farm in the Almaty region (Kazakhstan). The milk was obtained from Talgat local dairy farm located in Kargaly village, Zhambyl district, Almaty region, Kazakhstan. The milk was collected from animals under standard feeding conditions and exhibited naturally elevated fat content within the range occasionally observed in bovine milk depending on breed, lactation stage, and nutritional factors and was collected during a single production period.

For experimental consistency, milk was pooled into a single batch prior to processing. All subsequent analyses were performed in triplicate (*n* = 3), representing analytical replicates rather than independent biological batches.

### 2.2. Production of Skim Milk

Skim milk was obtained by centrifugation of pasteurized milk at 40–45 °C using a laboratory separator (Motor Sich 100, Motor Sich, Kyiv, Ukraine). Separation was performed under centrifugal force, yielding cream and skim milk. The residual fat content in skim milk did not exceed 0.1%. Skim milk was cooled to 4 ± 2 °C before further processing.

### 2.3. Production of Cottage Cheese Whey

Milk was standardized for fat content when necessary and pasteurized at 85–90 °C for 5–6 min. After cooling to 30 ± 2 °C, mesophilic starter cultures (1–5%) were added, until a pH of approximately 4.5–4.6 was reached.

Cottage cheese whey was produced using the acid coagulation method. Coagulation proceeded for 6–8 h until a stable curd was formed. Coagulation was determined based on both pH endpoint and visual gel stability. The process was conducted under controlled laboratory conditions with continuous monitoring of temperature and time. The curd was then cut into 2 × 2 cm cubes.

To enhance syneresis, the curd was heated at 36–38 °C for 15–20 min. Whey was separated by drainage and self-pressing.

The obtained cottage cheese whey was filtered and cooled to 4 °C.

### 2.4. Production of Cheese Whey

Milk was matured at 8–12 °C for 10–14 h prior to processing. Standardization was performed to optimize the fat-to-protein ratio.

Pasteurization was carried out at 63–65 °C for 20 min or 71–72 °C for 20–25 s. After cooling to 32–36 °C, starter culture (0.5–0.8%), CaCl_2_ (10–40 g/100 kg milk), and rennet enzyme (1:1000 dilution) were added.

Coagulation occurred within 20–30 min. The curd was cut, stirred, and partially drained. Whey was separated, filtered, and cooled to 4 °C.

### 2.5. Production of Yogurt Whey

Milk was standardized to a fat content of 1–3.5% and homogenized at 60–70 °C under a pressure of 15–25 MPa. Pasteurization was carried out at 90–95 °C for 5–10 min. After cooling to 40–43 °C, starter cultures consisting of *Streptococcus thermophilus* and *Lactobacillus delbrueckii* subsp. *bulgaricus* (2–5%, *w*/*v*) were inoculated. The mixture was then filled into retail containers and incubated at 40–43 °C for 3–6 h until the pH reached 4.4–4.6. Subsequently, the samples were cooled to 4–6 °C, and the whey released during syneresis was collected and filtered.

### 2.6. Storage Conditions

All whey samples (cottage cheese, cheese, and yogurt whey) were stored at 4 ± 2 °C for no longer than 48 h prior to analysis. For long-term storage, samples were frozen at −18 °C.

### 2.7. Pasteurization Conditions

Pasteurization of milk and dairy fractions was performed according to established dairy processing standards, with temperature–time regimes both selected based on industrial practice to ensure microbiological safety while preserving physicochemical and nutritional properties.

The applied thermal treatments were optimized considering the differential heat sensitivity of milk components. In particular, whey proteins are known to undergo denaturation at temperatures above 80–90 °C, whereas casein micelles exhibit higher thermal stability. Therefore, specific pasteurization conditions were selected depending on the type of dairy fraction and technological objective.

The following pasteurization regimes were applied:

- Raw milk (for general processing): 72–75 °C for 15–20 s (high-temperature short-time, HTST);
- Milk for cottage cheese production: 85–90 °C for 5–6 min;
- Milk for yogurt production: 90–95 °C for 5–10 min;
- Milk for cheese production: 63–65 °C for 20 min or 71–72 °C for 20–25 s;
- Whey fractions: 80–85 °C for 30–60 s.

These regimes ensure adequate inactivation of vegetative microorganisms and indicator enzymes, while minimizing excessive protein denaturation and maintaining functional properties of the dairy matrix.

All pasteurization processes were carried out under laboratory conditions using a computer-controlled processing unit (Edibon RDC, Computer Controlled Teaching Cottage Cheese Maker, Edibon International S.A., Madrid, Spain).

### 2.8. Determination of Physical and Chemical Properties

Immediately after collection, milk samples were placed in a portable refrigerator and transported to the laboratory. Upon arrival, samples were gently mixed to ensure homogeneity and stored at 4 °C until analysis, which was performed within 24 h. The contents of fat, protein, casein, lactose, solids-not-fat (SNF), total solids (TS), citric acid, urea, and acidity (°D) were determined by infrared spectroscopy using a MilkoScan FT1 analyzer (Foss Electric, Hillerød, Denmark). The instrument was factory-calibrated for mare milk and validated for bovine milk and whey matrices using standard calibration checks and comparative measurements. A minimum sample volume of 25 mL was required to perform duplicate analyses for each sample [26]. Although the instrument had previously been optimized in our laboratory for mare milk applications, this additional optimization was not used as the primary calibration for the current bovine milk and whey analyses. Prior to analysis, instrument performance was verified using routine calibration checks and reference control samples with known compositional ranges. Therefore, the physicochemical measurements of bovine milk and whey fractions were performed within the appropriate calibration framework for the studied matrices.

### 2.9. Amino Acid Determination

The analysis of amino acids (AAs) in milk was performed by high-performance liquid chromatography (HPLC) following acid hydrolysis and pre-column derivatization with phenyl isothiocyanate (PITC). For hydrolysis, 1000 µL of the milk sample was treated with 6 mol/L of HCl and incubated at 110 °C for 13 h in sealed tubes. The hydrolysates were then dried under vacuum to remove residual acid.

Derivatization was carried out by resuspending the dried residue in 150 µL of 0.1 mol/L NaOH, 50 µL of deionized water, and 350 µL of PITC reagent (propanol:PITC:triethylamine, 8:1:1, *v*/*v*/*v*). The reaction mixture was incubated at room temperature for 30 min, after which excess PITC was removed under a nitrogen stream. The derivatized samples were reconstituted in 1.5 mL of deionized water and filtered through a 0.45 µm membrane filter, and 10 µL was injected into the HPLC system.

Chromatographic analysis was performed using a Shimadzu Prominence LC-20 system (Shimadzu, Kyoto, Japan) equipped with a binary pump, autosampler, degasser, column oven, and UV detector, controlled by LCsolution software (version 1.26; Shimadzu Corporation, Kyoto, Japan). Separation was achieved on a Thermo Hypersil GOLD C18 column (150 × 4 mm, 5 µm). The mobile phase consisted of (A) acetonitrile with 1% acetic acid and (B) ultrapure water containing 0.1% acetic acid and 0.1 M sodium acetate. The flow rate was set at 0.8 mL/min, with a total run time of 43 min, and detection was carried out at 254 nm.

External calibration was performed using amino acid standards (purity ≥ 99%, Titan Biotech Ltd., New Delhi, India), including aspartic acid, glutamic acid, serine, asparagine, histidine, arginine, threonine, alanine, proline, cysteine, tyrosine, valine, methionine, cystine, isoleucine, leucine, phenylalanine, and lysine. Stock solutions (1 mg/mL) were prepared in 6 mol/L of HCl, evaporated, and derivatized as described above. Calibration curves were constructed at five concentration levels (1–100 µg/mL) [27].

The chromatographic parameters, calibration curves, and analytical performance characteristics, including linearity (R^2^), limits of detection (LODs), and limits of quantification (LOQs), for all analyzed amino acids are presented in Table S1. Representative chromatograms illustrating amino acid separation are shown in Figure S1. It should be noted that tryptophan was not determined in this study, as it is degraded during acid hydrolysis with hydrochloric acid. Therefore, the reported amino acid composition does not include tryptophan.

### 2.10. Desirability Calculation

The individual responses were normalized to a 0–1 scale using min–max normalization. Nutritional quality (NQ) was calculated based on protein, casein, TS, SNF, ΣEAA, and ΣBCAA, which were set to be maximized. Functional balance (FB) was defined based on optimal intermediate values of lactose and acidity-related parameters. Sustainability score (SS) was assigned proportionally to whey content, reflecting the increasing valorization of dairy by-products.

The overall desirability (D) was calculated as the weighted geometric mean:

$$D=(NQ^{w_{1}}\cdot FB^{w_{2}}\cdot SS^{w_{3}}{)}^{1/(w_{1}+w_{2}+w_{3})}$$

where equal weights (w_1_ = w_2_ = w_3_ = 1) were applied.

### 2.11. Preparation of Whey–Milk Mixtures

**Figure 1:**
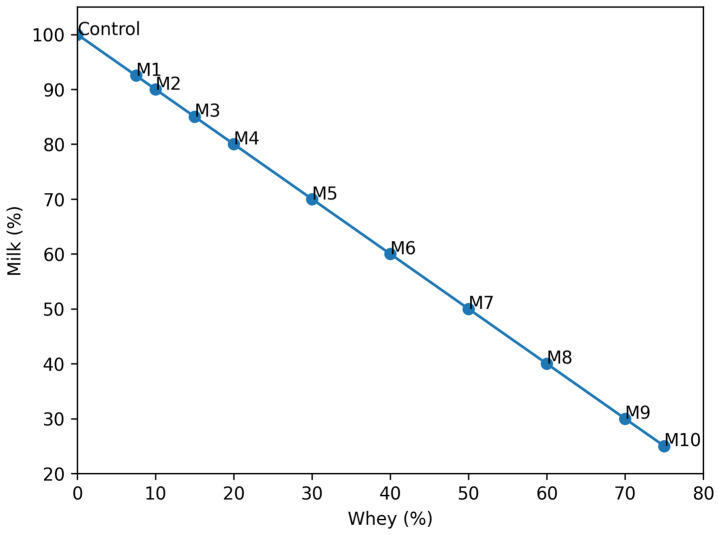
Composition of milk–whey mixtures (M1–M10) expressed as percentage ratios of milk and whey.

Model systems were prepared by adding whole cow’s milk to fresh bovine whey in increasing proportions (*v*/*v*), as shown in Table 1 and Figure 1. Ten mixtures were formulated to evaluate the effect of milk addition level on the physicochemical and compositional parameters of whey-based systems.

The milk inclusion levels were: 7.5%, 10%, 15%, 20%, 30%, 40%, 50%, 60%, 70%, and 75% (*v*/*v*).

The remaining fraction consisted of bovine whey. All mixtures were prepared under constant stirring at 20 ± 2 °C to ensure homogeneity prior to analysis. Mixtures were prepared by combining milk and whey in defined proportions, expressed as percentage contributions of each component (milk % and whey %, *v*/*v*), ensuring clear distinction between phases.

### 2.12. Statistical Analysis

All experiments were performed in triplicate (*n* = 3), and the results are presented as mean ± standard deviation.

Statistical analysis was carried out using JMP PRO 17. One-way analysis of variance (ANOVA) was used to evaluate differences among sample groups, including (i) milk and whey product types and (ii) milk–whey mixture formulations (M1–M10), depending on the dataset, followed by Tukey’s honestly significant difference (HSD) post hoc test to determine pairwise comparisons. Differences were considered statistically significant at *p* < 0.05. Replicates (*n* = 3) represent analytical replicates performed from the same pooled sample batch rather than independent biological replicates.

Multivariate statistical analysis, including principal component analysis (PCA) and hierarchical cluster analysis (HCA), was performed to assess relationships among physicochemical parameters and amino acid profiles. PCA was used to reduce dimensionality and identify the main sources of variation, while HCA was conducted using Ward’s linkage method with column standardization to visualize sample clustering.

Pearson correlation analysis was applied to evaluate relationships between physicochemical parameters and amino acid fractions, and the results are presented as a correlation heatmap.

## 3. Results

### 3.1. Physicochemical Characteristics of Milk and Whey Products

Whole milk exhibited significantly higher fat content (6.26%) compared with all other products (*p* < 0.05), whereas skim milk and all whey fractions showed similarly low fat levels (0.15–0.17%) and did not differ significantly from each other. A similar pattern was observed for total solids (TS) and casein. Milk demonstrated the highest TS (15.70%) and casein (2.90%), followed by skim milk (9.81% and 3.54%, respectively), while whey products showed significantly lower values (*p* < 0.05), reflecting removal of fat and casein during processing, as shown in Table 2.

Protein content also differed significantly among products. Milk contained the highest protein level (3.83%), followed by skim milk (3.54%). Among whey fractions, cottage whey (1.34%) had significantly higher protein than cheese whey (1.02%) and yogurt whey (0.70%) (*p* < 0.05). This indicates varying degrees of protein retention depending on processing technology.

SNF values were highest in skim milk (9.66%) and milk (9.44%), which differed significantly from all whey samples (6.71–7.31%). Lactose concentration showed an opposite trend: cheese whey exhibited the highest lactose content (5.54%), followed by cottage whey (5.22%) and yogurt whey (5.14%), while milk had the lowest lactose level (4.87%) (*p* < 0.05). This confirms lactose enrichment in whey following casein removal.

Acidity-related parameters further differentiated the products. Titratable acidity (°D) and lactic acid content were significantly elevated in fermented whey fractions. Cottage whey showed the highest acidity and lactic acid concentration, followed by yogurt whey, whereas milk had the lowest values (*p* < 0.05). These findings reflect fermentation-induced biochemical transformations. Although a strong proportional relationship was observed between titratable acidity (°D) and lactic acid content across the analyzed samples, it should be noted that Dornic acidity reflects total titratable acidity rather than lactic acid exclusively. In addition to lactic acid, other components—including citric acid, phosphate salts, dissolved carbon dioxide, and protein buffering systems—also contribute to the overall acidity of dairy matrices. The observed correspondence is therefore primarily due to the dominant role of lactic acid in fermented products, whereas the contribution of other acidic components remains relatively constant and comparatively minor.

Freezing point values differed significantly among all products, with cheese whey showing the most negative value, indicating higher soluble solid concentration. Density was highest in skim milk and lowest in yogurt whey, suggesting compositional dilution and redistribution of solids.

Urea levels varied markedly, with cottage whey showing the highest concentration, while cheese whey exhibited the lowest (*p* < 0.05).

Overall, Tukey’s test confirmed that milk is compositionally distinct due to its intact fat–protein matrix, skim milk is characterized by fat removal but retention of protein and SNF, and whey products are primarily differentiated by lactose enrichment, reduced casein, and increased acidity-related parameters. These results demonstrate clear technological and biochemical differentiation among dairy matrices.

**Figure 2:**
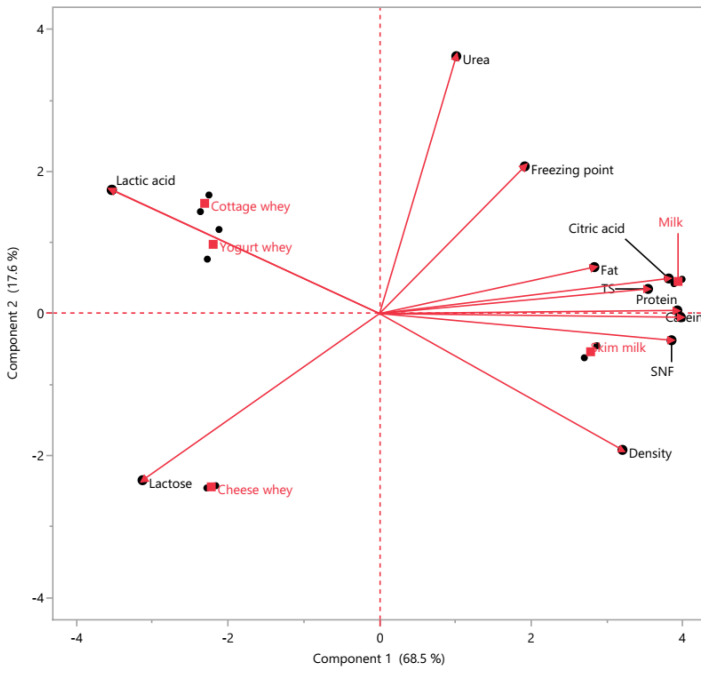
PCA biplot showing multivariate differentiation of milk and whey products based on physicochemical parameters.

Principal component analysis (PCA) demonstrated a clear compositional differentiation between milk and whey products (Figure 2). The first two principal components explained 86.1% of the total variance, with PC1 and PC2 accounting for 68.5% and 17.6%, respectively, indicating that most of the physicochemical variability can be effectively represented in a two-dimensional space.

PC1 primarily separated whole milk from whey-derived products. Positive loadings on PC1 were strongly associated with fat, total solids (TS), protein, SNF, citric acid, and density, all of which clustered closely, indicating strong positive intercorrelations. Samples located on the positive side of PC1, particularly milk, were characterized by higher structural macronutrient content and overall solids concentration. In contrast, negative PC1 loadings were associated mainly with lactose and acidity-related parameters, positioning whey products—especially cheese whey—on the opposite side of the axis. Thus, PC1 reflects a compositional gradient from intact milk matrix to whey fractions resulting from protein and fat removal.

PC2 provided additional discrimination among whey subtypes. Positive PC2 loadings were mainly associated with urea and freezing point, while negative loadings were related to density and lactose. This secondary axis reflects biochemical and mineral-related differences, particularly those influenced by fermentation and processing conditions. Yogurt whey clustered in the region associated with higher acidity, consistent with fermentation-driven transformations, whereas cheese whey was closely aligned with lactose content. Cottage whey occupied an intermediate position, reflecting mixed compositional characteristics. Skim milk clustered near milk along PC1 but shifted along PC2 due to reduced fat content.

PCA revealed a clear differentiation of dairy matrices based on physicochemical composition (Tables S2 and S3). PC1, explaining the largest variance, was positively associated with protein, casein, SNF, TS, density, and citric acid, and negatively with acidity, lactic acid, and lactose, separating milk and skim milk (positive scores) from all whey types (negative scores). PC2 was mainly influenced by urea and freezing point, distinguishing yogurt and cottage whey from cheese whey. PC3 was driven primarily by fat (negative) and freezing point (positive), differentiating whole milk from skim milk. Overall, PCA effectively discriminated milk and whey matrices, with PC1 representing the main compositional contrast and PC2–PC3 capturing secondary variations (Figure S2).

Overall, the PCA biplot demonstrates strong multivariate clustering according to product type and confirms substantial physicochemical differentiation among milk and whey products. The clear separation and vector orientation indicate that fat–protein–solid fractions drive the primary compositional variability, while fermentation-related and osmotic parameters contribute to secondary differentiation.

### 3.2. Amino Acid Profile and Nutritional Quality

The amino acid composition of milk and dairy products is summarized in Table 3. Significant differences (*p* < 0.05) were observed among products for all analyzed amino acids. Fresh milk exhibited the highest concentrations of both individual amino acids and total amino acid fractions. In particular, glutamic acid and aspartic acid were the predominant amino acids in all samples, reaching 6.98 ± 0.21 and 4.62 ± 0.14 g/100 g protein in milk, respectively. These amino acids are key components of casein proteins and play an important role in maintaining protein structure and supporting metabolic processes. A similar dominance of glutamic and aspartic acids has been reported previously for bovine and other mammalian milk proteins.

One-way ANOVA followed by Tukey’s HSD test (*p* < 0.05) revealed significant differences among milk and whey products for all analyzed parameters.

Fermented products such as yogurt and cheese demonstrated significantly lower absolute amino acid concentrations compared with milk (*p* < 0.05). This decrease is primarily associated with technological processes including fermentation, protein coagulation, and moisture redistribution during processing. During lactic fermentation, microbial proteolytic systems partially hydrolyze casein proteins, generating peptides and free amino acids that may subsequently be utilized by lactic acid bacteria for growth and metabolic activity. As a result, the measured concentrations of intact amino acids in yogurt and cheese were lower than those observed in fresh milk. Among fermented products, cheese showed the lowest values for most amino acids, which may reflect the removal of soluble nitrogen fractions with whey during curd formation.

Cottage cheese whey and skim milk showed intermediate amino acid levels, significantly higher than those of yogurt and cheese but lower than fresh milk (*p* < 0.05). The relatively elevated amino acid content in cottage cheese whey may be attributed to the retention of a large portion of milk proteins during curd formation combined with partial concentration effects.

The sum of essential amino acids (ΣEAAs) followed a similar pattern, with the highest value observed in milk (8.02 ± 0.22 g/100 g protein), followed by cottage cheese whey (3.50 ± 0.11 g/100 g protein) and skim milk (3.04 ± 0.10 g/100 g protein). Fermented products showed significantly lower EAA levels, particularly cheese (1.79 ± 0.06 g/100 g protein). Essential amino acids are critical for human nutrition because they cannot be synthesized endogenously and must be obtained through dietary sources. The high EAA content in milk and cottage cheese whey therefore highlights their nutritional importance as high-quality protein sources.

Particular attention should be given to branched-chain amino acids (ΣBCAAs: leucine, isoleucine, and valine), which play a central role in muscle protein synthesis, metabolic regulation, and energy metabolism. In this study, total ΣBCAAs were highest in milk (4.32 ± 0.14 g/100 g protein) and cottage cheese (1.87 ± 0.07 g/100 g protein), whereas lower concentrations were observed in yogurt (1.02 ± 0.04 g/100 g protein) and cheese (0.95 ± 0.04 g/100 g protein). BCAAs are known to activate the mechanistic target of rapamycin (mTOR) signaling pathway, thereby supporting muscle protein synthesis and maintenance, particularly in physically active individuals and the elderly. Therefore, dairy products with higher BCAA concentrations may provide additional nutritional benefits.

The total content of ΣNEAA also differed significantly among products (*p* < 0.05), with the highest concentration observed in milk (20.86 ± 0.63 g/100 g protein). These amino acids contribute to metabolic homeostasis and serve as precursors for numerous biologically active compounds.

Overall, the results demonstrate that milk retains the highest amino acid concentration among the studied dairy products, while fermentation and processing lead to significant reductions in amino acid levels. Nevertheless, fermented dairy products remain valuable sources of EAA and BCAA, supporting their nutritional relevance in human diets.

### 3.3. Multivariate Correlation and Clustering Analysis of Physicochemical and Amino Acid Profiles

Hierarchical cluster analysis (HCA), performed using Ward’s linkage method with column standardization, revealed clear multivariate differentiation between milk and whey-derived products based on their amino acid composition. The dendrogram demonstrated two primary clusters corresponding to (i) whole milk and (ii) whey fractions, indicating substantial compositional restructuring following dairy processing.

Whole milk samples formed a distinct and compact cluster, reflecting consistently higher concentrations of all amino acids. The tight grouping of milk replicates confirms strong analytical reproducibility and compositional homogeneity. In contrast, whey products clustered separately, demonstrating significantly reduced amino acid levels due to casein removal during cheese and yogurt production.

**Figure 3:**
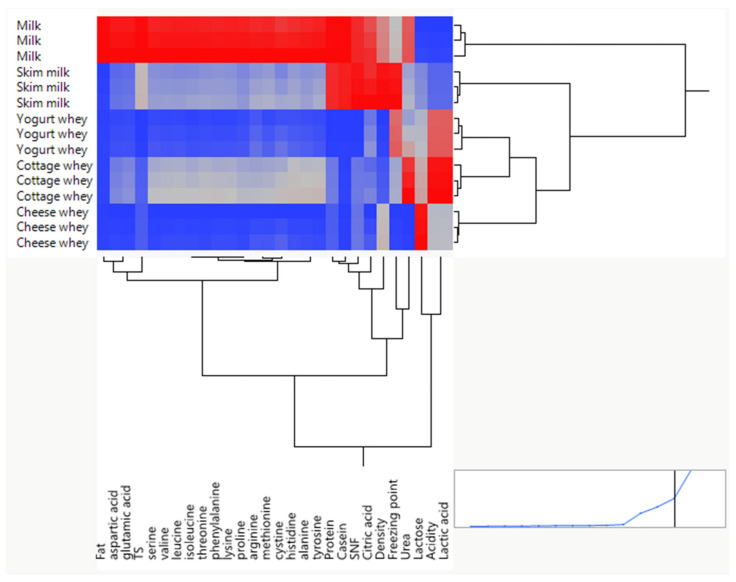
Multivariate hierarchical clustering revealing compositional differentiation between milk and whey products based on amino acid profiles and physicochemical characteristics. Color intensity represents relative standardized values of the analyzed variables, where red corresponds to higher values and blue corresponds to lower values.

Within the whey cluster, further sub-structuring was observed. Yogurt whey and cheese whey grouped closely together, indicating highly similar amino acid patterns characterized by uniformly low concentrations across both essential (EAA) and non-essential amino acids (NEAA). This similarity reflects comparable protein depletion following coagulation and whey separation processes. Cottage whey formed a partially distinct branch, suggesting intermediate amino acid retention relative to other whey fractions. This intermediate positioning likely reflects differences in production technology and protein partitioning. Skim milk occupied a transitional position between whole milk and whey products, consistent with fat removal while largely preserving casein-associated amino acids (Figure 3).

Column clustering of variables demonstrated strong grouping among branched-chain amino acids (valine, leucine, isoleucine), acidic amino acids (aspartic and glutamic acid), and other essential amino acids. The high degree of co-clustering confirms strong intercorrelation and preservation of the intrinsic milk protein amino acid structure across all products. No distinct clustering of individual amino acids unique to specific whey types was observed, indicating that technological processing primarily reduces overall amino acid concentration rather than selectively modifying proportional amino acid distribution.

Overall, the clustering analysis confirms that milk is compositionally distinct due to its intact protein matrix, whereas whey products represent protein-depleted derivatives with highly similar relative amino acid patterns. The separation between milk and whey is therefore driven mainly by the quantitative reduction in amino acids rather than qualitative changes in protein structure. These findings support the conclusion that dairy processing alters total protein content but largely preserves the intrinsic amino acid signature of bovine milk proteins.

**Figure 4:**
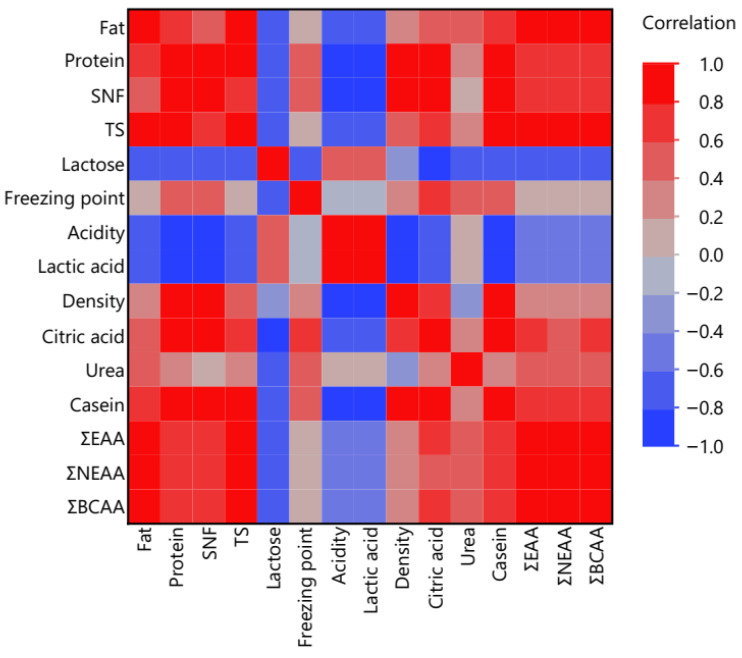
Correlation heatmap of physicochemical parameters and amino acid fractions in dairy samples.

The relationships between physicochemical parameters and amino acid fractions in dairy samples were assessed using a Pearson correlation matrix, visualized as a heatmap (Figure 4). The analysis revealed several strong positive correlations among protein-related variables and amino acid fractions.

In particular, protein, casein, TS, and SNF exhibited strong positive correlations with ΣEAA, ΣNEAA, and ΣBCAA, indicating that samples with higher protein content generally contained higher concentrations of both essential and non-essential amino acids. This relationship reflects the fact that amino acids constitute the fundamental structural units of milk proteins, particularly casein, which represents the dominant protein fraction in dairy products.

A very strong positive correlation was also observed between ΣEAA and ΣBCAA, which is expected because branched-chain amino acids (leucine, isoleucine, and valine) represent a major subset of essential amino acids. Similarly, ΣNEAA showed strong associations with protein, SNF, and casein content, further confirming the dependence of amino acid composition on total protein concentration.

Conversely, lactose displayed negative correlations with several protein-related variables and amino acid fractions, suggesting that samples with higher lactose levels tended to contain lower concentrations of protein-derived compounds. This inverse relationship is commonly observed in dairy matrices due to compositional dilution effects between carbohydrate and protein fractions.

Acidity and lactic acid were positively correlated with each other but showed weaker or negative relationships with several protein variables. This pattern likely reflects the biochemical transformations occurring during fermentation processes, where microbial metabolism converts lactose into lactic acid while simultaneously modifying protein structures.

Moderate correlations were also observed between citric acid and certain physicochemical parameters, indicating complex interactions between lipid metabolism, organic acid production, and protein composition during dairy processing.

Overall, the correlation analysis demonstrates that protein-associated parameters (protein, casein, SNF, and TS) are the primary drivers of amino acid distribution in dairy products, while carbohydrate- and fermentation-related variables contribute to compositional variability among dairy matrices. These relationships highlight the interconnected nature of physicochemical composition and amino acid profiles in dairy systems.

### 3.4. Integrated Desirability Function Analysis and Optimization

**Figure 5:**
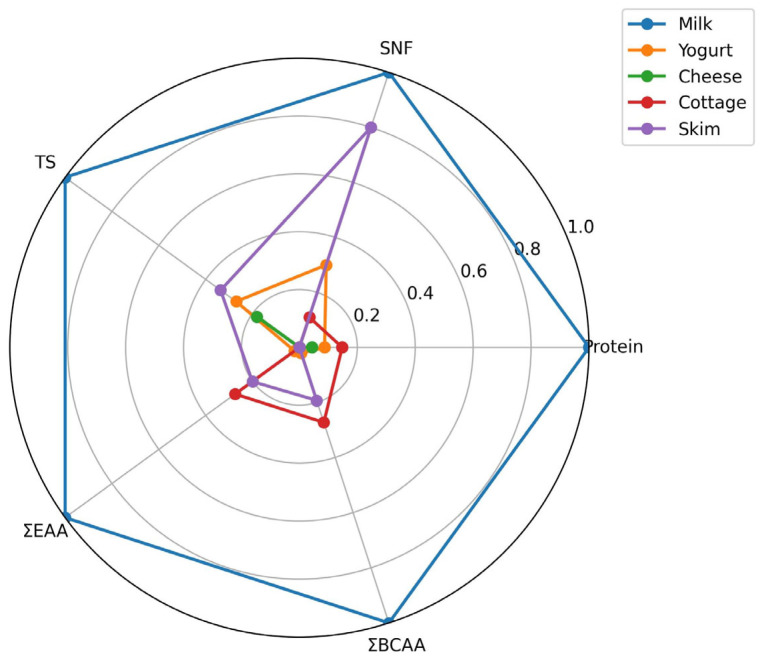
Radar plot comparing dairy products based on normalized physicochemical and amino acid quality indicators.

To identify the most promising whey-derived matrix after combining physicochemical and amino acid data, an overall desirability function was applied using the measured responses from Table 2 and Table 3. In this multi-response optimization, nutritionally favorable variables such as protein, total solids, SNF, ΣEAA, ΣBCAA, and total amino acids were set to be maximized, whereas less desirable indicators associated with compositional depletion were minimized or constrained within acceptable ranges. Based on this integrated approach (Figure 5), cottage whey was selected as the optimal product among the whey-based samples, because it consistently showed the most advantageous balance between compositional concentration and nutritional value. Among whey fractions, cottage whey had the highest protein content (1.34%), the highest total solids (7.48%), and one of the highest SNF values (7.31%), indicating superior retention of nutritionally relevant solids. In addition, cottage-derived samples exhibited markedly higher amino acid levels than yogurt and cheese counterparts, including ΣEAA (3.50), ΣNEAA (6.80), and ΣBCAA (1.87), confirming its superior protein quality. Although whole milk remained highest for most traits overall, the desirability-based optimization specifically aimed to identify the best whey-derived system, and under this criterion, cottage whey achieved the most favorable overall response profile. Therefore, cottage whey can be considered the most suitable raw material for further formulation of value-added whey–milk systems due to its superior combined physicochemical and amino acid characteristics.

Based on the comparative analysis of physicochemical properties and amino acid composition among the evaluated whey fractions, cottage cheese whey was identified as the most suitable matrix for further formulation. Therefore, all subsequent milk–whey mixtures (M1–M10) used in the optimization analysis were prepared exclusively using cottage cheese whey.

### 3.5. Integrated Multivariate Analysis and Quality Evaluation of Milk–Whey Mixtures

PCA was conducted to examine the relationships among physicochemical parameters, amino acid composition, and sample distribution. The first two principal components explained 92.8% of the total variance, with PC1 and PC2 accounting for 50.5% and 42.3%, respectively, indicating that the dataset is well represented in a two-dimensional space.

**Figure 6:**
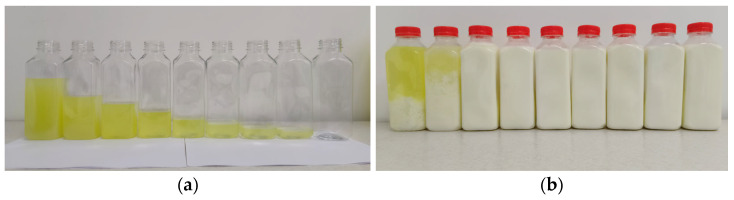
Integrated visual representation of milk–whey mixtures across formulations: (**a**) milk–whey mixtures before processing; (**b**) milk–whey mixtures after processing.

PC1 (52.7%) was primarily associated with nutritional and compositional variables. Strong positive loadings were observed for protein, fat, TS, SNF, casein, ΣEAA, and ΣBCAA, indicating that these variables are highly correlated and represent the major axis of compositional richness. Samples M1–M4 and M5 were located on the positive side of PC1, suggesting higher nutritional density, particularly in terms of protein fractions and essential amino acids. In contrast, samples M7–M10, which shown in Figure 6, were located on the negative side of PC1, indicating comparatively lower values of these parameters.

**Figure 7:**
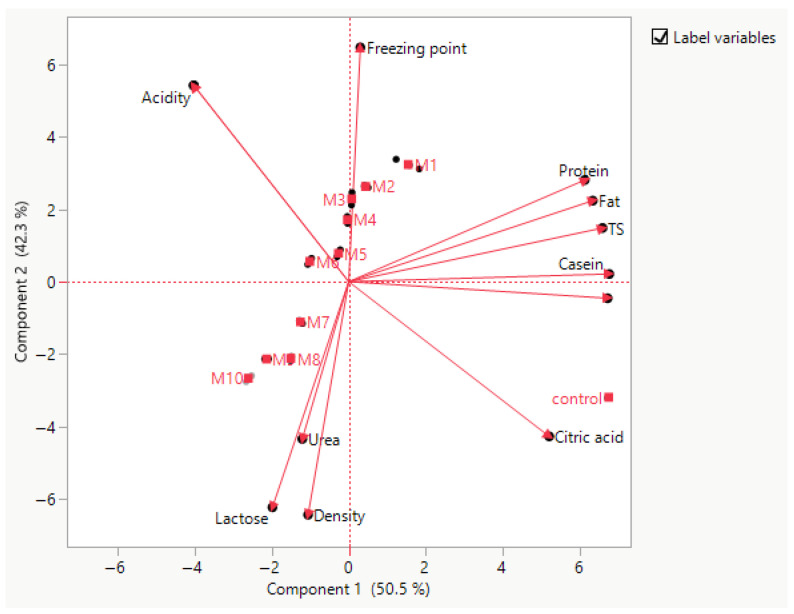
PCA biplot of physicochemical properties and amino acid parameters of milk–whey mixtures.

PC2 (35.2%) was mainly influenced by acidity-related and minor compositional factors. Negative loadings were associated with acidity, lactic acid, and freezing point, while positive loadings corresponded to lactose, density, and urea. This suggests that PC2 differentiates samples based on freshness, fermentation-related changes, and physicochemical stability. Samples M2–M4 were distributed in the negative PC2 region, indicating higher acidity and lactic acid content, whereas samples such as M8–M10 showed positive PC2 values, associated with higher lactose and density (Figure 7).

The control sample was distinctly separated in the upper-right quadrant, strongly correlated with citric acid and SNF, indicating a unique compositional profile compared to experimental samples.

Overall, the PCA clearly demonstrates that samples M1–M4 represent nutritionally superior profiles (high protein, casein, ΣEAA, ΣBCAA), while samples M7–M10 are characterized by higher lactose-related parameters and lower protein content. The separation along PC2 further highlights the influence of acidity and fermentation-related changes on sample differentiation.

**Figure 8:**
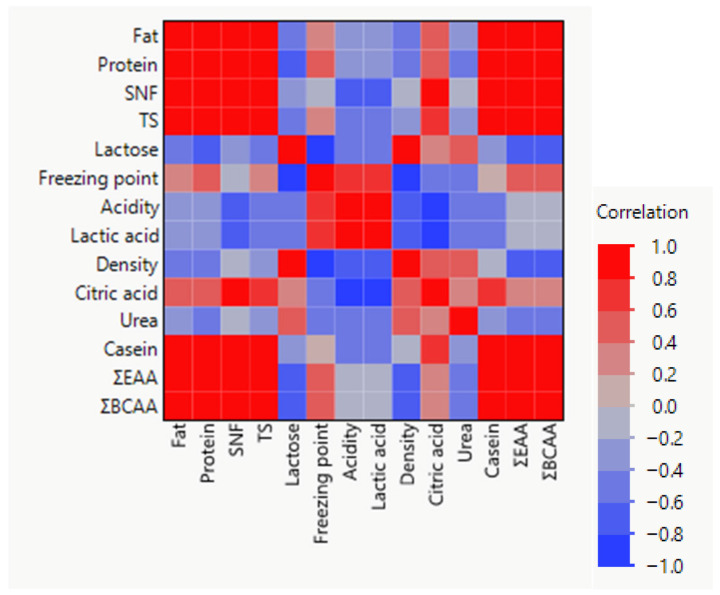
Correlation heatmap of physicochemical parameters and amino acid fractions in milk-whey mixtures.

The Pearson correlation analysis based on functional food samples (M1–M10) and the control revealed a well-structured relationship between compositional and physicochemical parameters, reflecting formulation-driven differences, shown in Figure 8.

A strong positive correlation network was observed among protein, fat, SNF, TS, casein, ΣEAA, and ΣBCAA, confirming that these parameters collectively represent the nutritional and protein-enriched matrix of the functional products. Notably, ΣEAA and ΣBCAA were highly correlated with total protein and casein, indicating that enrichment strategies directly enhanced essential amino acid content across the formulations.

Conversely, lactose showed significant negative correlations with protein-related variables, suggesting a dilution or substitution effect in functional formulations, where increased protein enrichment corresponds to reduced carbohydrate (lactose) proportion. This pattern clearly differentiates protein-fortified samples from more carbohydrate-dominant formulations.

The acidity and lactic acid parameters formed a strong positive correlation pair, representing the fermentation-related characteristics of the products. These variables were generally negatively associated with density and partially with protein fractions, indicating biochemical changes linked to fermentation intensity among samples.

Freezing point demonstrated moderate positive correlations with lactose and citric acid, while showing inverse relationships with acidity, consistent with the influence of soluble solids and organic acids on colligative properties.

Density and citric acid occupied an intermediate position, showing moderate correlations with both compositional and acidity-related variables, suggesting their role as integrative indicators of product structure and stability.

In contrast, urea displayed weaker and less consistent correlations with other parameters, indicating that these components are influenced by specific metabolic or processing factors rather than overall formulation composition.

These findings confirm that protein enrichment is the primary driver of compositional variability among M1–M10 samples, while lactose and acidity contribute to secondary differentiation linked to formulation and fermentation processes, distinguishing functional products from the control.

### 3.6. Integrated PCA–Desirability Analysis for Optimal Mixture Selection

The PCA biplot showed that the first two principal components explained 87.9% of the total variance (PC1 = 52.7%, PC2 = 35.2%), indicating effective representation of formulation differences in two-dimensional space. PC1 was positively associated with protein, casein, TS, SNF, ΣEAA, and ΣBCAA—variables defining the NQ component—suggesting that samples with higher PC1 scores exhibited superior nutritional profiles. Conversely, samples with negative PC1 scores were characterized by lower levels of these components and reduced nutritional desirability.

The second principal component (PC2) further separated the samples according to lactose, acidity, lactic acid, density, and freezing point, which are directly related to the FB domain. Samples with strongly negative PC2 scores were associated with higher acidity and lactic acid, whereas those with positive PC2 values were more related to lactose and density. This distribution suggests that the most desirable formulations were not necessarily those located at the extremes of PC2, but rather those occupying positions that reflected a more balanced physicochemical profile. Thus, the integrated PCA interpretation supports the desirability concept that optimal mixtures should simultaneously combine high nutritional density with moderate and technologically acceptable acidity-related characteristics.

The inclusion of the SS adds a formulation-development dimension that is not directly visible in PCA alone. Since SS increased proportionally with whey content, mixtures with greater incorporation of whey-derived ingredients gained an additional advantage in the final overall desirability value. Therefore, the best-performing mixture was expected to be the one that achieved a favorable compromise among all three domains: high protein and amino acid enrichment (high NQ), balanced lactose and acidity properties (high FB), and increased utilization of dairy by-products (high SS).

Overall, the integrated PCA–desirability approach demonstrated that optimal mixture selection was driven primarily by the variables contributing to nutritional enrichment along PC1, while physicochemical balance along PC2 refined the selection, and sustainability further strengthened the preference for whey-based formulations. This combined statistical framework provides a robust and scientifically justified basis for identifying the most suitable functional food mixture, as it accounts simultaneously for composition, technological balance, and sustainability-related value.

The calculated desirability values revealed clear differences among the functional food formulations. The nutritional quality (NQ) was highest in samples M1–M4, reflecting their strong association with the protein, casein, and amino acid variables identified in PCA. In contrast, later formulations (M7–M10) showed lower NQ values, consistent with reduced protein enrichment and higher lactose-related composition (Table 4).

The functional balance (FB) values were more evenly distributed across samples, with slightly higher values observed in M5–M8, indicating improved balance between lactose and acidity-related parameters. This suggests that moderate formulations achieved better physicochemical stability compared to extreme compositions.

The sustainability score (SS) increased progressively from M1 to M10, reflecting the higher incorporation of whey-based components in later formulations. This trend confirms that sustainability is inversely related to protein concentration but positively associated with by-product utilization.

The integrated desirability (D) identified M5 (D = 0.78) as the optimal formulation, representing the best compromise between high nutritional quality, balanced physicochemical properties, and enhanced sustainability. Although samples M1–M3 exhibited superior NQ values, their lower sustainability scores reduced their overall desirability. Conversely, samples M8–M10 showed high sustainability but insufficient nutritional quality.

The superior performance of the M5 formulation (30% whey) can be explained by the optimal balance between casein-rich milk components and whey-derived proteins and lactose. At lower whey inclusion levels (M1–M4), the system remains dominated by milk constituents, resulting in higher casein content but limited contribution from whey proteins, which restricts improvements in amino acid diversity and sustainability metrics. Conversely, at higher whey proportions (M6–M10), dilution of casein and total solids becomes more pronounced, leading to reduced protein concentration, weaker structural integrity, and less favorable physicochemical properties.

At the intermediate level represented by M5, a synergistic interaction between casein micelles and whey proteins is achieved, which supports both nutritional quality and functional performance. This balance is reflected in relatively high values of protein, total solids, and essential amino acids (ΣEAA and ΣBCAA), while maintaining acceptable lactose levels and acidity. In addition, from a sustainability perspective, the incorporation of 30% whey represents a meaningful valorization of dairy by-products without compromising product quality.

PCA further supports this interpretation, as M5 is positioned in a region associated with favorable loadings of protein-related variables while avoiding the negative effects linked to excessive whey inclusion. Similarly, the desirability function integrates these competing factors and identifies M5 as the formulation that achieves the most balanced compromise among nutritional, functional, and sustainability criteria. Therefore, the optimality of M5 reflects a trade-off between compositional enrichment and structural stability, rather than maximization of a single parameter.

Overall, the desirability analysis confirms the PCA interpretation and demonstrates that optimal formulation is achieved through a balance between protein enrichment and whey utilization rather than maximizing a single parameter.

## 4. Discussion

The physicochemical analysis confirmed clear compositional distinctions among whole milk, skim milk, and the three whey fractions, consistent with their respective production technologies. Whole milk exhibited significantly higher fat content (6.26%), total solids (15.70%), protein (3.83%), and casein (2.90%) compared with all whey products (*p* < 0.05), reflecting the intact fat–protein matrix that is retained prior to any processing step. These results are consistent with previous studies indicating that milk is the most compositionally complete dairy matrix, whereas whey fractions represent protein- and fat-reduced derivatives formed during coagulation and whey separation [28,29].

Among whey types, cottage cheese whey demonstrated the highest protein content (1.34%), total solids (7.48%), and SNF (7.31%), as well as the highest titratable acidity (28.69 °D) and lactic acid (0.287%), reflecting the intensified fermentation conditions associated with acid-coagulated cottage cheese whey production. Cheese whey, produced under rennet-coagulated conditions at lower fermentation intensities, showed the highest lactose concentration (5.54%) and most negative freezing point (−0.604 °C), indicating greater soluble solid retention in this fraction [30].

The lactose gradient observed across products—increasing progressively from whole milk (4.87%) through skim milk (5.04%) to cheese whey (5.54%)—is consistent with the well-established principle that casein removal during curd formation concentrates the carbohydrate fraction in the residual whey serum. The strong negative correlations observed between lactose and protein-related parameters in the Pearson correlation analysis further confirm this compositional dilution mechanism. A similar inverse relationship between lactose and protein fractions has been reported in multivariate analyses of dairy matrices [31,32].

The amino acid composition of dairy products reflected the degree of protein retention following processing. Whole milk exhibited the highest concentrations of all individual amino acids, with glutamic acid (6.98 ± 0.21 g/100 g protein) and aspartic acid (4.62 ± 0.14 g/100 g protein) as the predominant fractions, consistent with their major structural role in casein micelles. The dominance of glutamic and aspartic acids in bovine milk protein fractions has been widely reported and reflects the amino acid composition of αs1-casein and β-casein, which together constitute the majority of the casein fraction [32].

Fermented products (yogurt and cheese) demonstrated significantly lower amino acid concentrations compared with fresh milk (*p* < 0.05), with the lowest values recorded for cheese (ΣEAA = 1.79 ± 0.06 g/100 g protein; ΣBCAA = 0.95 ± 0.04 g/100 g protein). This reduction can be attributed to multiple concurrent processes: proteolytic hydrolysis by lactic acid bacteria generates soluble peptides and free amino acids that are subsequently catabolized during fermentation, and the partitioning of soluble nitrogen fractions with the whey phase during curd formation removes a significant proportion of total amino acids from the protein matrix. These observations align with established mechanisms of microbial proteolytic activity in fermented dairy systems [33].

Particular attention is warranted for the BCAA fraction. The ΣBCAA values were highest in whole milk (4.32 ± 0.14 g/100 g protein) and cottage cheese whey (1.87 ± 0.07 g/100 g protein), substantially exceeding those of yogurt whey (1.02 ± 0.04 g/100 g protein) and cheese whey (0.95 ± 0.04 g/100 g protein). BCAAs—leucine, isoleucine, and valine—are well documented as key regulators of muscle protein synthesis through activation of the mechanistic target of rapamycin (mTOR) signaling pathway. In particular, leucine functions as the primary anabolic trigger for mTORC1 phosphorylation, stimulating downstream targets including S6K1 and 4E-BP1, thereby enhancing mRNA translation efficiency and net muscle protein accretion. The elevated BCAA content of cottage whey relative to other whey fractions therefore confers a distinct nutritional advantage in formulations targeting protein quality and muscle health outcomes [34,35,36].

The PCA and correlation analyses provided a useful overview of compositional relationships among physicochemical and amino acid variables; however, these results should be interpreted primarily as descriptive rather than mechanistic. The observed clustering patterns and correlations largely reflect expected relationships inherent to dairy systems, such as the positive association between protein- and casein-related parameters and amino acid fractions, and the inverse relationship with lactose due to compositional dilution effects. While these multivariate approaches effectively summarize data structure and support the differentiation of milk and whey-derived products, they do not, by themselves, establish causal relationships or underlying biochemical mechanisms. Therefore, the results should be interpreted within the context of compositional analysis rather than mechanistic inference.

The PCA of milk–whey mixtures, explaining 87.9% of total variance (PC1 = 52.7%; PC2 = 35.2%), demonstrated that samples M1–M5 were positioned on the positive PC1 axis, strongly associated with protein, casein, fat, ΣEAA, and ΣBCAA, while samples M7–M10 occupied the negative PC1 region, reflecting their lower protein content and higher lactose-related parameters. This distribution confirms that the primary axis of variability in milk–whey mixtures corresponds to nutritional density, in agreement with the PCA structure observed for the initial milk and whey product characterization. The modification of whey-to-casein ratio in dairy systems has been documented to substantially alter protein composition and physicochemical behavior, including heat stability, viscosity, and gel-forming capacity [37,38].

The correlation heatmap of mixture samples revealed a strong positive network among protein, fat, SNF, TS, casein, ΣEAA, and ΣBCAA, confirming the coordinated enrichment of nutritional parameters in formulations with higher milk proportions. The strong negative correlation between lactose and protein-related variables observed across mixtures further supports the compositional substitution mechanism: as milk content increases relative to whey, protein enrichment occurs at the cost of proportional lactose reduction. These correlation patterns are consistent with findings from studies on functional dairy formulations where protein fortification systematically alters carbohydrate composition [39].

The application of the desirability function to the initial characterization data identified cottage cheese whey as the most suitable raw material among whey fractions for use in functional dairy formulations. Cottage whey achieved the highest combined score across nutritional quality indicators, including the highest ΣEAA (3.50 g/100 g protein), ΣBCAA (1.87 g/100 g protein), total solids (7.48%), and protein content (1.34%) among whey products. These compositional advantages reflect the retention of whey proteins and partial proteolysis products during acid-mediated cottage cheese whey production, which preserves a greater fraction of nutritionally relevant protein compounds in the serum phase compared with rennet-coagulated cheese processing [32,40].

The elevated protein and amino acid content of cottage whey relative to yogurt and cheese whey can be explained by differences in coagulation mechanism. In acid coagulation, casein precipitation occurs at the isoelectric point (pH 4.6), leaving whey proteins largely intact in solution. In rennet coagulation, κ-casein cleavage initiates micelle aggregation under conditions that may more efficiently retain soluble proteins within the curd network, resulting in lower residual protein in cheese whey. Cottage whey’s higher acidity (28.69 °D) relative to cheese whey (0.242 mmol/kg; 20.70 °D) reflects the more intense fermentation environment, which, while increasing acidity-related parameters, also contributes to the partial hydrolysis of proteins that may enhance bioavailability. Previous studies have demonstrated that cottage cheese whey proteins exhibit high antioxidant activity upon enzymatic hydrolysis, further supporting its valorization potential as a functional food ingredient [40,41].

The desirability function analysis of the ten milk–whey mixture formulations identified M5 (30% whey, 70% milk) as the optimal formulation, achieving the highest overall desirability score (D = 0.78) among all tested systems. This result reflects the specific balance achieved by M5 across the three composite dimensions: nutritional quality (NQ = 0.80), functional balance (FB = 0.78), and sustainability score (SS = 0.75). The integrated analysis demonstrates that compositional maximization alone—as represented by M1 (NQ = 0.92)—is insufficient as a formulation criterion when sustainability and physicochemical balance are simultaneously considered.

The NQ component, based on protein, casein, TS, SNF, ΣEAA, and ΣBCAA, showed the highest values in low-whey formulations (M1–M4), consistent with the PCA loading structure where PC1 positive values were associated with these variables. However, the SS, which increased proportionally with whey content, reflecting a greater valorization of dairy by-products, penalized these protein-rich formulations in the overall desirability calculation. High-whey formulations (M7–M10) achieved maximal SSs (0.82–0.86) but demonstrated insufficient NQ values (0.50–0.58), confirming that extreme whey incorporation compromises protein quality beyond acceptable thresholds. This trade-off between nutritional density and sustainability is a fundamental challenge in the formulation of whey-enriched dairy products and has been documented in functional beverage and yogurt optimization studies [38,42].

Formulation M5 achieved an optimal compromise by incorporating sufficient whey content to generate a meaningful sustainability contribution while maintaining protein and amino acid levels above the critical threshold at which nutritional quality becomes limiting. The FB score of M5 (0.78), reflecting optimal intermediate lactose and acidity parameters, further supports its technological suitability, as excessively high acidity from whey-dominant formulations may pose challenges for heat stability and sensory acceptability in dairy applications. The ability to incorporate up to 30% whey without substantial loss of nutritional functionality represents a practically significant finding, consistent with reports that controlled whey incorporation in dairy beverages and functional drinks can enhance mineral density and maintain acceptable physicochemical properties [6,39].

The present findings have direct relevance to circular bioeconomy frameworks in the dairy sector. Global whey generation exceeds 115 million tonnes annually, with approximately 47% discharged as wastewater, contributing a biochemical oxygen demand (BOD) of 40–60 g/L and chemical oxygen demand (COD) of 50–80 g/L to receiving water bodies. Regulatory pressures in the European Union and other jurisdictions have intensified the urgency of valorizing whey as a value-added ingredient rather than an environmental liability. The formulation of whey–milk mixtures at the M5 composition represents a scalable strategy for incorporating substantial quantities of whey-derived ingredients into nutritionally functional dairy products without requiring intensive downstream processing such as membrane fractionation or spray drying [43,44].

The sustainability score integrated into the desirability function operationalizes circular bioeconomy principles within a multi-criteria optimization framework, providing a quantitative basis for selecting formulations that simultaneously achieve nutritional adequacy and resource efficiency. This approach extends beyond conventional food product development paradigms that optimize single or few response variables, addressing the multi-dimensional nature of sustainable food system design. Cottage whey, identified as the optimal raw material for such formulations, offers an additional processing advantage in that it is generated from acid-coagulated systems that do not require rennet addition, simplifying the valorization pathway for small-scale dairy producers [45].

This study has several limitations that should be considered when interpreting the results. Tryptophan was not quantified, due to degradation during acid hydrolysis, and therefore the reported EAA content represents a partial profile. In addition, the identification of the M5 formulation (30% whey) as the most favorable is based on the applied desirability model and selected variables, and does not account for sensory properties, storage stability, rheological behavior, or bioavailability.

The desirability function approach itself involves a degree of subjectivity, particularly in the selection of variables and the application of equal weighting, which may influence the ranking of formulations. The relatively small differences observed among mixtures further indicate that several formulations may be considered comparably suitable depending on specific objectives.

Finally, the study was conducted using bovine milk and whey from a single production context. Variability related to breed, season, feeding, and processing conditions, as well as differences in non-bovine milk systems, were not addressed and may influence the generalizability of the findings.

## 5. Conclusions

This study demonstrated that the composition and functional properties of dairy systems can be effectively modulated by controlled incorporation of whey into milk. Significant differences were observed among milk and whey fractions, with whey-derived products characterized by lower protein and casein content but enriched in lactose and acidity-related components. Among the evaluated whey types, cottage cheese whey exhibited the most favorable balance of physicochemical and amino acid characteristics, making it a suitable candidate for further formulation.

Multivariate analysis confirmed that protein-related parameters (protein, casein, total solids, and amino acid fractions) are the primary drivers of compositional variability, while fermentation-related factors contribute to secondary differentiation. The integration of these variables through desirability function analysis enabled the identification of optimal formulations based on multiple criteria rather than single-parameter maximization.

Among the tested formulations, the mixture containing 30% whey (M5) showed the highest desirability within the applied model, reflecting a balanced combination of nutritional, physicochemical, and sustainability-related parameters. This result highlights that moderate whey incorporation can enhance resource utilization without compromising product performance.

However, this finding should be interpreted within the limitations of the applied model and selected variables. The optimization does not account for sensory quality, storage stability, rheological properties, or bioavailability, and therefore does not represent a definitive optimal product in a practical context. Future research should focus on validating these findings through sensory evaluation, functional and rheological characterization, shelf-life studies, and assessment of nutrient bioavailability. Extension of the proposed approach to different milk sources and processing conditions, as well as refinement of the optimization model through alternative weighting strategies, would further strengthen its applicability in dairy product development.

Overall, the findings support the application of integrated analytical and optimization approaches for the development of value-added dairy systems. The proposed framework can be used to guide the rational design of whey-enriched products within the context of sustainable food production and circular bioeconomy strategies.

## Figures and Tables

**Table 1:** Composition of experimental whey–milk mixtures (*v*/*v*).

Sample	Whey (%)	Milk (%)
Control	0	100
M1	7.5	92.5
M2	10	90
M3	15	85
M4	20	80
M5	30	70
M6	40	60
M7	50	50
M8	60	40
M9	70	30
M10	75	25

**Table 2:** Physicochemical characteristics of milk and dairy whey fractions.

Parameter	Milk	Skim Milk	Yogurt Whey	Cheese Whey	Cottage Whey
Fat (%)	6.26 ± 0.03 ^a^	0.15 ± 0.03 ^b^	0.16 ± 0.01 ^b^	0.15 ± 0.01 ^b^	0.17 ± 0.01 ^b^
Protein (%)	3.83 ± 0.01 ^a^	3.54 ± 0.02 ^b^	0.70 ± 0.01 ^e^	1.02 ± 0.02 ^d^	1.34 ± 0.02 ^c^
SNF (%)	9.44 ± 0.02 ^b^	9.66 ± 0.05 ^a^	6.71 ± 0.01 ^e^	7.28 ± 0.02 ^d^	7.31 ± 0.02 ^c^
TS (%)	15.70 ± 0.05 ^a^	9.81 ± 0.08 ^b^	6.87 ± 0.02 ^e^	7.43 ± 0.03 ^d^	7.48 ± 0.03 ^c^
Lactose (%)	4.87 ± 0.01 ^e^	5.04 ± 0.01 ^d^	5.14 ± 0.01 ^c^	5.54 ± 0.01 ^a^	5.22 ± 0.01 ^b^
Freezing point (°C)	−0.569 ± 0.001 ^c^	−0.544 ± 0.001 ^e^	−0.556 ± 0.001 ^d^	−0.604 ± 0.001 ^a^	−0.576 ± 0.001 ^b^
Acidity °Dornic (°D)	14.35 ± 0.17 ^e^	16.35 ± 0.09 ^d^	25.37 ± 0.09 ^b^	20.70 ± 0.05 ^c^	28.69 ± 0.21 ^a^
Lactic acid (%)	0.143 ± 0.002 ^e^	0.163 ± 0.001 ^d^	0.254 ± 0.001 ^b^	0.207 ± 0.001 ^c^	0.287 ± 0.002 ^a^
Density (g/L)	1029.0 ± 0.3 ^c^	1034.8 ± 0.2 ^a^	1019.0 ± 0.2 ^e^	1026.2 ± 0.2 ^d^	1020.2 ± 0.2 ^b^
Citric acid (%)	0.18 ± 0.01 ^b^	0.20 ± 0.01 ^a^	0.06 ± 0.01 ^c^	0.02 ± 0.01 ^d^	0.05 ± 0.01 ^c^
Urea (mg/dL)	519.6 ± 2.6 ^b^	414.0 ± 12.2 ^c^	430.5 ± 32.5 ^c^	278.1 ± 1.4 ^d^	560.3 ± 15.6 ^a^
Casein (%)	2.90 ± 0.01 ^a^	2.72 ± 0.02 ^b^	0.05 ± 0.01 ^d^	0.06 ± 0.01 ^c^	0.08 ± 0.01 ^e^

Values were analyzed using one-way analysis of variance (ANOVA) followed by Tukey’s HSD post hoc test. Data are expressed as mean ± standard deviation (*n* = 3). Different lowercase letters within the same row indicate statistically significant differences between samples according to Tukey’s HSD post hoc test at *p* < 0.05.

**Table 3:** Amino acid composition of milk and dairy products (g/100 g protein).

Amino Acid	Milk	Yogurt	Cheese	Cottage Cheese	Skim Milk
Aspartic acid	4.62 ± 0.14	0.66 ± 0.03	0.58 ± 0.03	1.08 ± 0.05	0.92 ± 0.04
Glutamic acid	6.98 ± 0.21	1.12 ± 0.05	0.98 ± 0.04	1.86 ± 0.08	1.55 ± 0.07
Serine	1.46 ± 0.06	0.35 ± 0.02	0.31 ± 0.01	0.62 ± 0.03	0.54 ± 0.02
Histidine	0.58 ± 0.03	0.19 ± 0.01	0.17 ± 0.01	0.31 ± 0.01	0.27 ± 0.01
Arginine	0.44 ± 0.02	0.15 ± 0.01	0.13 ± 0.01	0.21 ± 0.01	0.20 ± 0.01
Threonine	1.02 ± 0.04	0.24 ± 0.01	0.22 ± 0.01	0.45 ± 0.02	0.39 ± 0.02
Alanine	1.36 ± 0.06	0.41 ± 0.02	0.37 ± 0.02	0.69 ± 0.03	0.58 ± 0.02
Proline	2.12 ± 0.08	0.52 ± 0.02	0.47 ± 0.02	0.88 ± 0.04	0.76 ± 0.03
Tyrosine	0.86 ± 0.03	0.22 ± 0.01	0.20 ± 0.01	0.42 ± 0.02	0.36 ± 0.02
Valine	1.78 ± 0.06	0.42 ± 0.02	0.39 ± 0.02	0.77 ± 0.03	0.66 ± 0.03
Methionine	0.46 ± 0.02	0.13 ± 0.01	0.12 ± 0.01	0.21 ± 0.01	0.20 ± 0.01
Cystine	0.31 ± 0.02	0.11 ± 0.01	0.10 ± 0.01	0.16 ± 0.01	0.14 ± 0.01
Isoleucine	0.92 ± 0.03	0.21 ± 0.01	0.20 ± 0.01	0.39 ± 0.02	0.34 ± 0.02
Leucine	1.62 ± 0.05	0.39 ± 0.02	0.36 ± 0.02	0.71 ± 0.03	0.60 ± 0.03
Phenylalanine	0.74 ± 0.03	0.18 ± 0.01	0.17 ± 0.01	0.33 ± 0.02	0.29 ± 0.01
Lysine	1.46 ± 0.05	0.35 ± 0.02	0.33 ± 0.02	0.64 ± 0.03	0.56 ± 0.02
ΣEAA	8.02 ± 0.22	1.92 ± 0.07	1.79 ± 0.06	3.50 ± 0.11	3.04 ± 0.10
ΣNEAA	20.86 ± 0.63	4.05 ± 0.14	3.59 ± 0.12	6.80 ± 0.23	5.67 ± 0.19
ΣBCAA	4.32 ± 0.14	1.02 ± 0.04	0.95 ± 0.04	1.87 ± 0.07	1.60 ± 0.06

EAA—essential amino acid, NEAA—non-essential amino acid, BCAA—branched-chain amino acid (valine, leucine, isoleucine). Values represent mean of triplicate measurements (*n* = 3).

**Table 4:** Integrated evaluation of milk–whey mixtures based on nutritional quality, functional balance, sustainability score, and overall desirability (D).

Sample	NQ	FB	SS	Overall D
M1	0.92	0.71	0.65	0.75
M2	0.88	0.69	0.68	0.74
M3	0.83	0.72	0.72	0.75
M4	0.85	0.74	0.70	0.76
M5	0.80	0.78	0.75	0.78
M6	0.62	0.81	0.78	0.73
M7	0.55	0.76	0.82	0.70
M8	0.58	0.79	0.80	0.72
M9	0.52	0.77	0.84	0.71
M10	0.50	0.75	0.86	0.70

NQ—nutritional quality; FB—functional balance, SS—sustainability score; Overall D—overall desirability.

## Data Availability

The original contributions presented in this study are included in the article/Supplementary Materials. Further inquiries can be directed towards the corresponding author.

## Supplementary Materials

The following supporting information can be downloaded at https://www.mdpi.com/article/10.3390/foods15101759/s1, Figure S1: HPLC chromatogram of amino acid mix standard, analyzed on a Thermo Hypersil GOLD C18 HPLC column (150 mm × 4 mm, 5 µm), with UV detection at 254 nm; Figure S2: Principal component loadings of physicochemical variables and dairy matrices on the first three principal components; Table S1: Chromatographic parameters, calibration characteristics, and sensitivity (LOD and LOQ) for amino acid determination; Table S2. Principal component loadings of physicochemical parameters of milk samples; Table S3. Principal component scores of dairy matrices based on physicochemical properties.

## Abbreviations

The following abbreviations are used in this manuscript:

AA	Amino acids
EAA	Essential amino acids
NEAA	Non-essential amino acids
BCAA	Branched-chain amino acids
TS	Total solids
SNF	Solids-not-fat
HPLC	High-performance liquid chromatography
PCA	Principal component analysis
HCA	Hierarchical cluster analysis
NQ	Nutritional quality
FB	Functional balance
SS	Sustainability score
