# Supplementary material for: Integrated Multivariate Analysis and Desirability-Based Optimization of Milk–Whey Mixtures: Effects on Physicochemical Properties, Amino Acid Profile, and Nutritional Quality
Source: Foods. 2026 May 15;15(10):1759. doi: 10.3390/foods15101759 (PMC13206031; doi:10.3390/foods15101759)
Supplement: Supplementary file 1 [file foods-15-01759-s001.zip › foods-4273646-supplementary.pdf]

Supplementary materials

Calibration curves for all analyzed AAs were constructed using standard solutions within the concentration range of 1–100 μg/mL. As presented in Table S1, the peak area of each AA standard was plotted against its concentration, and linearity parameters were determined through linear regression analysis. The coefficient of determination (R^2) values were ≥0.995 for all AA compounds, indicating a high degree of linearity. The LOD and LOQ were assessed through progressive dilution of standard solutions. The obtained LOD values ranged from 0.03 μg/mL to 0.16 μg/mL, while LOQ values varied between 0.10 μg/mL and 0.47 μg/mL for all analyzed AAs, demonstrating the method's high sensitivity. The AA method’s accuracy and precision were validated through repeated analysis of the prepared amino acid mix standard, with retention times and peaks shown in Figure S1.

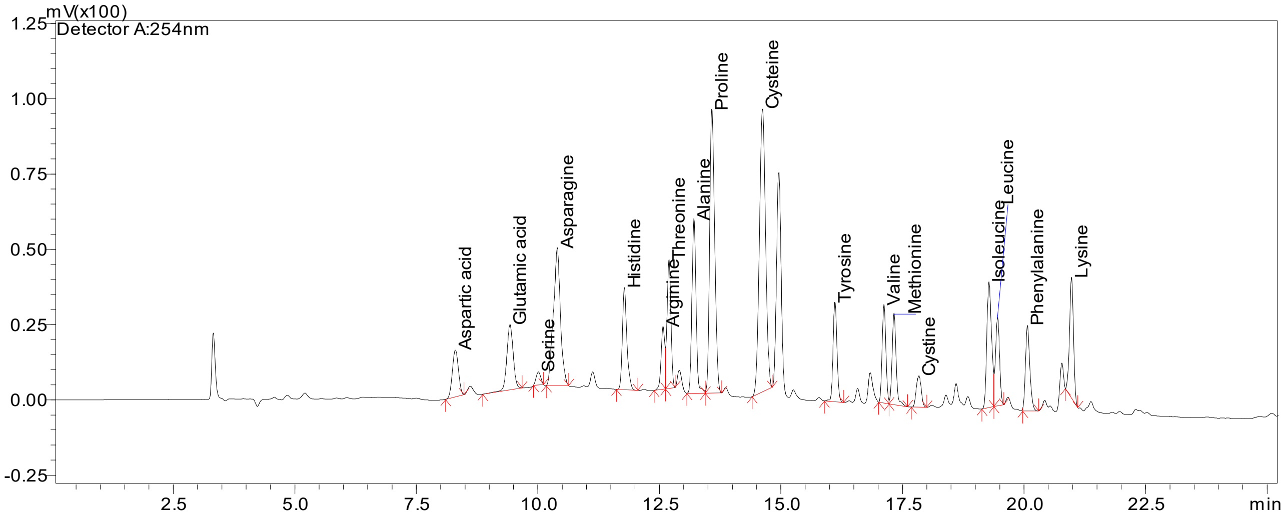

Figure S1. HPLC chromatogram of amino acid mix standard, analyzed on a Thermo Hypersil GOLD C18 HPLC column (150 mm × 4 mm, 5 μm), with UV detection at 254 nm.

Table S1. Chromatographic parameters, calibration characteristics, and sensitivity (LOD and LOQ) for amino acid determination.

#	Amino Acid	Retention time	R^2	Calibration Curve Equation	Calibration Range (μg/mL)	LOD (μg/mL)	LOQ (μg/mL)
1	Aspartic acid	8.387	0.9996	y = 1429.4x - 1421.1	1–100	0.06	0.19
2	Glutamic acid	9.486	0.9998	y = 2015.3x - 1076.4	1–100	0.03	0.10
3	Serine	10.417	0.9979	y = 3084.3x - 9055.9	1–100	0.15	0.46
4	Asparagine	10.427	0.9977	y = 1816.8x + 4740.6	1–100	0.16	0.47
5	Histidine	11.803	0.9997	y = 2305.8x - 2791.1	1–100	0.06	0.17
6	Arginine	12.596	0.9997	y = 1170.1x - 1494.1	1–100	0.06	0.18
7	Threonine	12.712	0.9998	y = 2538.2x - 3107.3	1–100	0.04	0.13
8	Alanine	13.224	0.9989	y = 3568.1x + 4727	1–100	0.05	0.14
9	Proline	13.589	0.9995	y = 7214.1x + 1226.3	1–100	0.07	0.21
10	Tyrosine	16.117	0.9997	y = 1904.6x + 1495.3	1–100	0.10	0.32
11	Valine	17.124	0.9991	y = 1858.6x + 2927.6	1–100	0.06	0.17
12	Methionine	17.331	0.9985	y = 1812.4x + 294.6	1–100	0.13	0.38
13	Cystine	17.842	0.9995	y = 783.1x + 1292.6	1–100	0.07	0.22
14	Isoleucine	19.284	0.9995	y = 3035.1x + 2905.7	1–100	0.07	0.22
15	Leucine	19.463	0.9985	y = 1717.1x + 447.11	1–100	0.13	0.39

16	Phenylalanine	20.076	0.9996	y = 1852.5x + 2184.7	1–100	0.06	0.20
17	Lysine	20.983	0.9996	y = 2401.7x + 3659.9	1–100	0.07	0.20

R—correlation coefficient; LOD—limit of detection; LOQ—limit of quantification.

Table S2. Principal component loadings of physicochemical parameters of milk samples.

	Prin1	Prin2	Prin3	Prin4	Prin5	Prin6	Prin7	Prin8	Prin9
Fat	0.70075	0.17778	-0.67144	-0.16188	0.01333	0.00261	0.01088	0.00115	0.00008
Protein	0.98539	0.05600	0.02601	0.15658	-0.01464	0.02101	-0.00414	-0.00360	0.00037
SNF	0.97283	-0.04611	0.13130	0.18322	-0.00404	0.02364	-0.00982	0.00168	0.00003
TS	0.88356	0.11511	-0.45071	-0.05224	0.00842	0.01074	0.00444	0.00148	0.00007
Lactose	-0.73940	-0.65704	0.00610	0.11712	0.08835	-0.00441	0.00102	0.00025	0.00071
Freezing point	0.43256	0.62735	0.62636	-0.16163	-0.02340	-0.01596	0.00865	0.00143	0.00041
Acidity	-0.90739	0.35743	0.02658	0.21775	-0.01256	0.02211	0.01029	0.00139	0.00117
Lactic acid	-0.90824	0.35673	0.02632	0.21536	-0.00988	0.02322	0.01151	0.00009	-0.00114
Density	0.83380	-0.42417	0.32448	0.13512	0.03583	0.00350	-0.00150	0.00277	-0.00060
Citric acid	0.94283	0.20667	0.24943	0.01747	0.07406	-0.00038	0.01861	-0.00202	-0.00017
FFA	-0.52389	0.79648	0.08433	-0.27989	0.07026	0.02319	-0.01523	0.00007	-0.00007
Urea	0.20398	0.86190	-0.25279	0.38703	0.02623	-0.03261	-0.00854	0.00019	-0.00005
Casein	0.99430	0.05513	0.08750	0.02219	-0.01164	0.00605	-0.00367	0.00032	0.00049

Table S3. Principal component scores of dairy matrices based on physicochemical properties.

	Prin1	Prin2	Prin3	Prin4	Prin5	Prin6	Prin7	Prin8	Prin9
Cheese whey	-1.91286	-2.91175	-0.45712	-0.07614	0.02360	-0.00238	-0.00030	0.00002	0.00002
Cottage whey	-2.42501	1.32538	-0.35509	1.02733	-0.01831	0.01050	-0.00042	-0.00010	-0.00005
Milk	3.94511	0.55920	-1.48418	-0.21572	0.00214	0.00013	0.00067	-0.00002	0.00000
Skim milk	2.86974	-0.38110	1.83351	0.25541	-0.00903	0.00059	-0.00098	0.00001	-0.00000
Yogurt whey	-2.47698	1.40826	0.46288	-0.99088	0.00160	-0.00884	0.00104	0.00009	0.00003

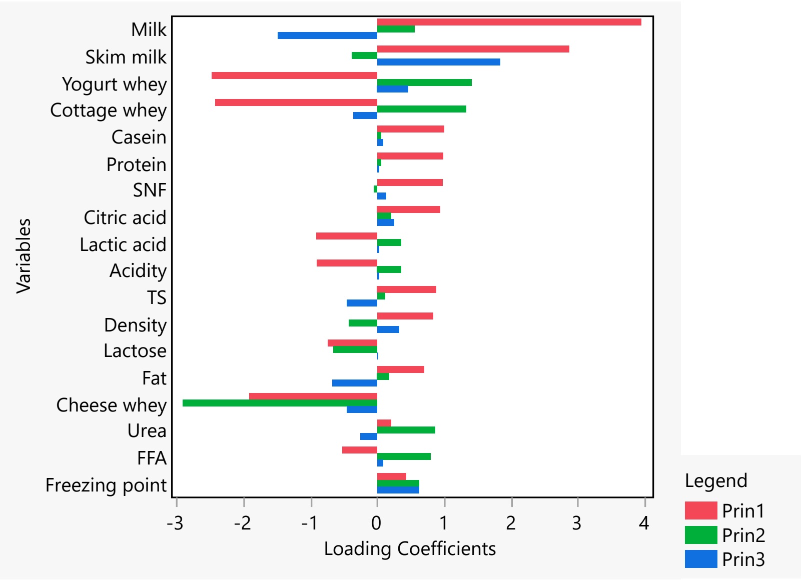

Figure S2. Principal component loadings of physicochemical variables and dairy matrices on the first three principal components.
